# A bibliometric analysis of light chain amyloidosis from 2005 to 2024: research trends and hot spots

**DOI:** 10.3389/fmed.2024.1441032

**Published:** 2024-07-30

**Authors:** Xiangdong Liu, Junyan Wang, Yingmin Li, Weibo Shi, Xiaojing Zhang, Shujin Li, Bin Cong

**Affiliations:** ^1^College of Forensic Medicine, Hebei Medical University, Shijiazhuang, China; ^2^Department of Oral and Maxillofacial Surgery, Hebei Key Laboratory of Stomatology, Hebei Clinical Research Center for Oral Diseases, Hebei Technology Innovation Center of Oral Health, School and Hospital of Stomatology, Hebei Medical University, Shijiazhuang, China; ^3^Postdoctoral Mobile Station of Basic Medical Science, Hebei Medical University, Shijiazhuang, China; ^4^Hebei Key Laboratory of Forensic Medicine, Hebei Collaborative Innovation Center of Forensic Medical Molecular Identification, Shijiazhuang, China; ^5^Research Unit of Digestive Tract Microecosystem Pharmacology and Toxicology, Chinese Academy of Medical Sciences, Shijiazhuang, China; ^6^Hainan Tropical Forensic Medicine Academician Workstation, Haikou, China

**Keywords:** AL amyloidosis, light chain amyloidosis, bibliometrics, research trend, Web of Science

## Abstract

**Background:**

Light chain (AL) amyloidosis stands as the most prevalent subtype of systemic amyloidosis, encompassing a group of rare diseases. Here, we evaluated the scientific landscape of AL amyloidosis to investigate research trends and identify hotspots within the field.

**Methods:**

Relevant studies on AL amyloidosis published over the past two decades were retrieved from the Web of Science Core Collection. The publications between 2005 and 2024 were subjected to bibliometric analyses, leveraging tools including CiteSpace, VOSviewer, RStudio and MS Excel to analyse and visualize the annual publication trend, co-occurrence patterns, collaborative networks among countries, organizations, and authors. Burst keywords and references were also examined to obtain the research history, and emerging hotspots.

**Results:**

The bibliometric analysis included 2,864 articles published between 2005 and 2024. The most productive journal is *Amyloid-Journal of Protein Folding Disorders*. The United States, along with several developed nations, emerges as a dominant force in international AL amyloidosis research. “AL amyloidosis” and “cardiac amyloidosis” were the primary hotspots over the past two decades, and “Biomarkers,” “Cardiac amyloidosis,” and “treatment” would be future trends.

**Conclusion:**

This bibliometric analysis examined the research developments in AL amyloidosis over the past two decades using bibliometric software. Recent research in this field primarily focuses on two main areas: clinical diagnosis and treatment of AL amyloidosis, as well as cardiac amyloidosis. Emphasis is placed on understanding the mechanisms underlying immunoglobulin light chain aggregation and deposition to mitigate organ involvement.

## 1 Introduction

Amyloidosis results from the extracellular deposition of amyloid, a fibril that self-assembles with highly ordered abnormal cross β-sheet conformation (1). There have been identified more than 60 proteins that are heterogeneous amyloidogenic and approximately more than 30 among them known to be associated with human disease (2). Immunoglobulin light chain (AL) amyloidosis or primary amyloidosis is the most frequent type of this rare group of diseases, with approximately 4,500 new cases diagnosed every year in the United States (3). It usually affects people from ages 50 to 80, with 64 years being the median age at diagnosis, and about two-thirds of the patients are male. AL amyloidosis is a clonal plasma cell disorder characterized by the systemic deposition of misfolded immunoglobulin light chains as insoluble fibrils or fragments in the extracellular space of various organs (4). The most common organ affected by AL amyloidosis is the heart, causing dysfunction and eventually death (5). Although significant advancements in the management and treatment of AL amyloidosis in the past 20 years, the disease remains fatal, especially in advanced patients (6). The molecular determinants of AL amyloidosis disease occurrence and development are still not fully clear (7).

Bibliometric analysis has recently emerged to be a popular and rigorous tool for investigating and analyzing large volumes of scientific literature (8). Using bibliometric analysis enables us to have a systematic and comprehensive understanding of the *de facto* structure in a particular field and capture emerging trends (9). Through measuring the scientific outputs of different scientific items, such as authors, keywords, journals, countries, and institutions, the results of the bibliometric method can be visualized in the form of figures and tables to get the research progress and identify the most contributed countries, authors, and institutions. As the most common form of systemic amyloidosis, it has entered a stage of rapid development, however, there is relatively few research in this field. To date, limited bibliometric studies have been conducted to describe the overall situation of AL amyloidosis. With the increasing number of literatures in this field, the bibliometric tool allows the researcher to identify the trends and status in research with an effective means.

In this study, we have conducted a comprehensive bibliometric analysis of AL amyloidosis during the period ranging from 2005 to 2024 using CiteSpace and VOSviewer, RStudio and MS Excel software. This review provides a systemic overview of the development trend and research outputs in the field of AL amyloidosis.

## 2 Materials and methods

### 2.1 Data sources

**Figure 1:**
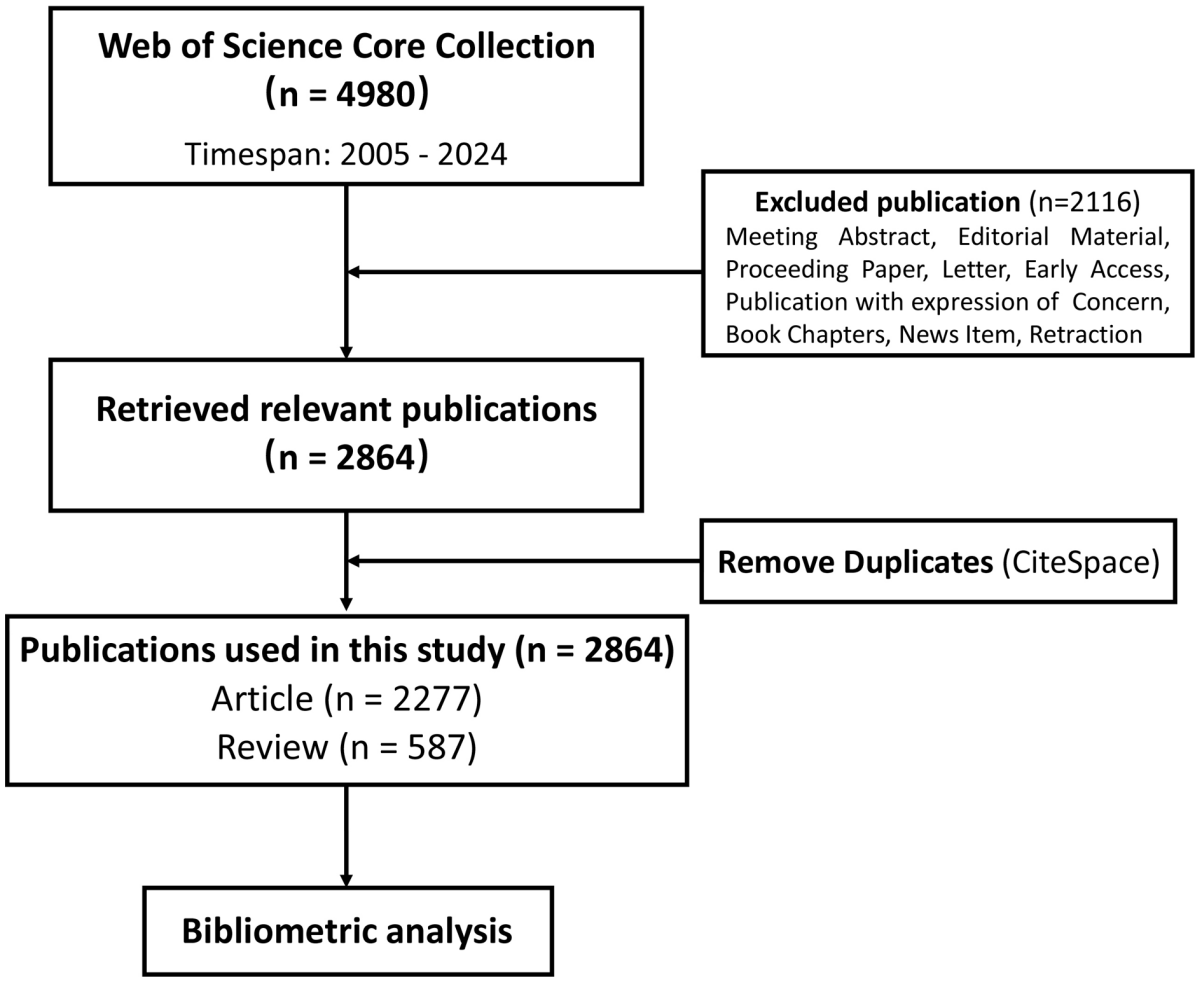
Search strategy.

Web of Science Core Collection (WOSCC) serves as a key global data source for literature searches. The current bibliometric study was conducted by searching relevant publications from the WOSCC database, which covers documents published from Jan 1, 2005 to Apr 28, 2024. The search strategy used in the database was as follows:

The search terms were set to TS = (“AL amyloidos?s” OR “Primary amyloidos?s” OR “light chain amyloidos?s” OR “amyloid light chain” OR “amyloidogenic light chain” OR “light chain aggregation” OR “Immunoglobulin light chain amyloidos?s”). “?” is a wildcard representing “i” or “e.” Data were retrieved on the 28th of April 2024. A total of 4,980 records were retrieved. Among these, we focus on relevant articles and reviews that are limited to the English language. Thus, 2,116 meeting abstract, editorial material, book reviews, proceeding paper, letter, early access, retraction, book chapters, news item, and publication with expression of concern were excluded, resulting in 2,864 documents published from 2005 to 2024. The results were exported and checked for duplicates using CiteSpace (version 6.3.R1). After checking duplicates (0 duplicates found), a total number of 2,864 publications including 2,277 articles and 587 reviews were obtained for further analysis. The search strategy is presented in Figure 1.

### 2.2 Data analysis

The present study employed CiteSpace (version 6.3.R1), VOSviewer (version 1.6.20), open-source Biblioshiny (R software, version 4.3.3), and MS Excel software for bibliometric analysis. CiteSpace is a web-based Java application for data analysis and visualization (10). The included documents were analyzed and visualized utilizing CiteSpace for keywords clusters, reference co-occurrence and top 25 keywords/references with bursts. The time-slicing specifications for CiteSpace were set to consider each year between 2005.01 and 2024.04 as a timestamp. VOSviewer is a software tool for creating and exploring maps based on network data (11). We used VOSviewer to explore keyword co-occurrence, author co-occurrence, country co-occurrence, and coupling analysis, including source journals and organizations. In addition, the *bibiometrix* (12)^1^ package of R software was used for the analysis of authors’ production over time, inter and intra collaboration of various countries. The Three-field plot analysis representing the author, country and source relationship was also conducted using *Biblioshiny*.

## 3 Results

### 3.1 Annual publications and growth trend

**Figure 2:**
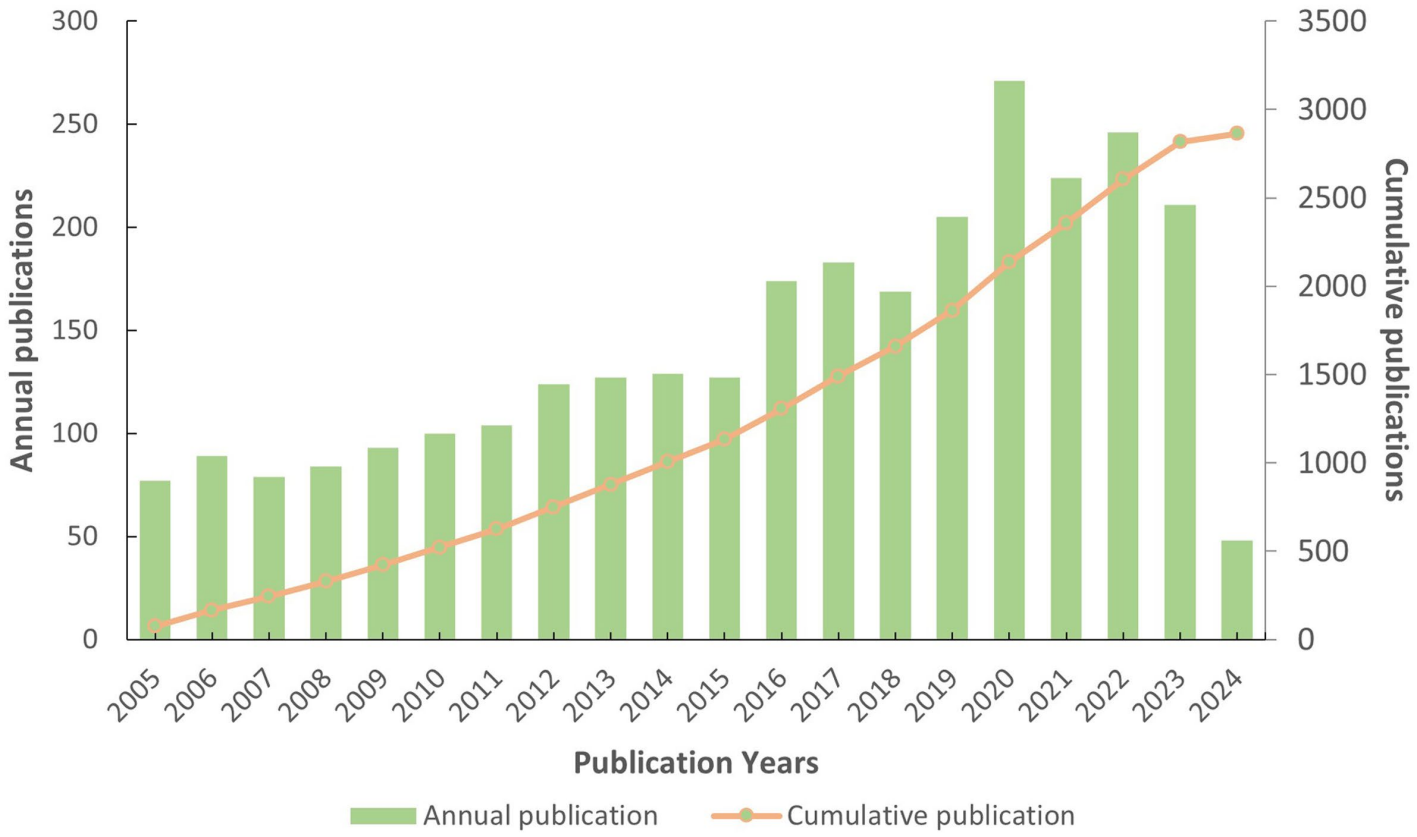
Yearly publication distribution and growth trend.

In the recent 20 years, a total of 2,864 articles in this field were published in the Web of Science. The volume of articles published in this field has generally shown a stable growth trend (Figure 2). The highest number of publications was published in 2020 (271/2,864, 9.462%).

### 3.2 Leading countries/regions and their collaboration

**Figure 3:**
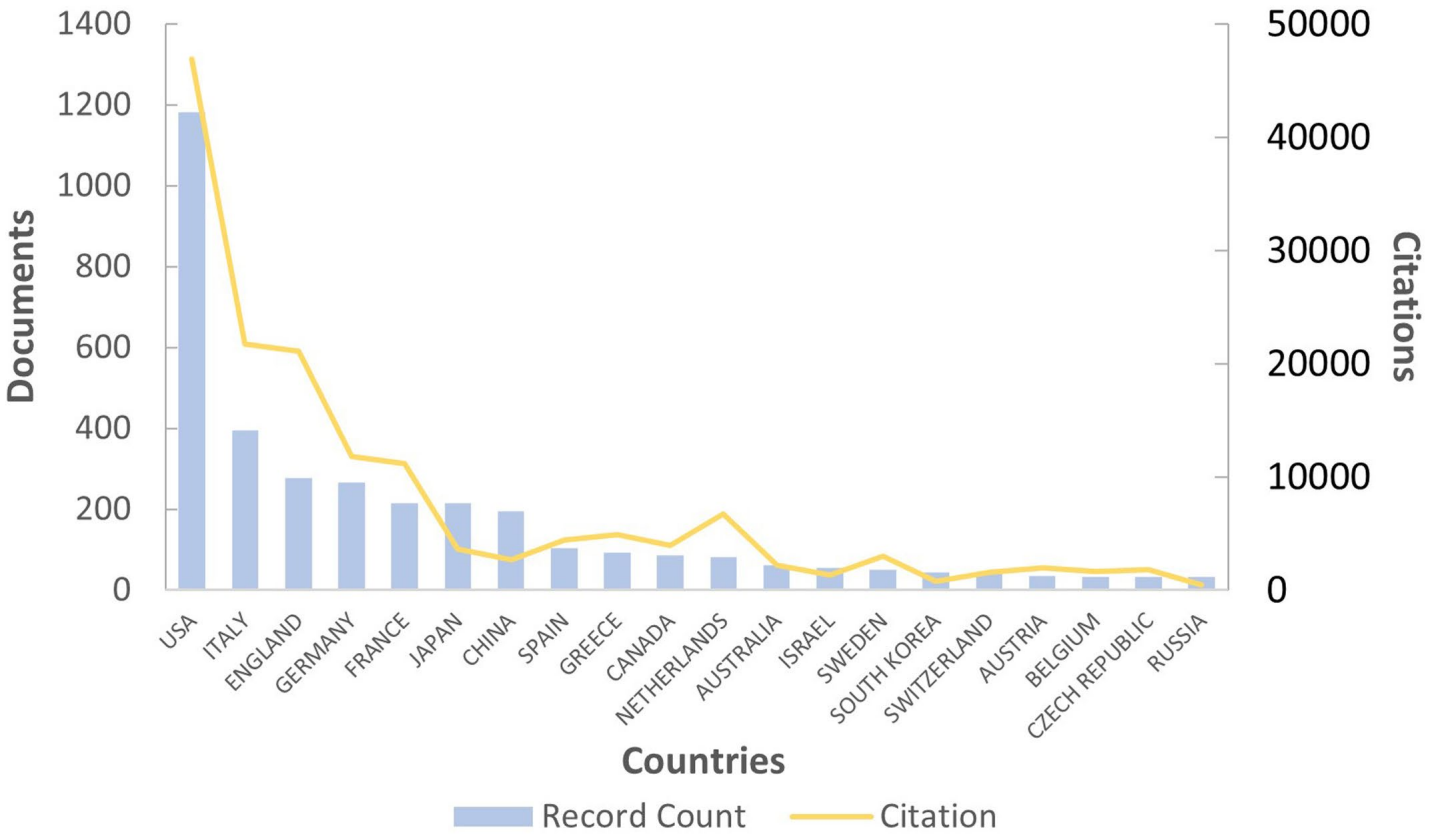
Top 20 countries with the highest number of papers and their citation trend.

**Figure 4:**
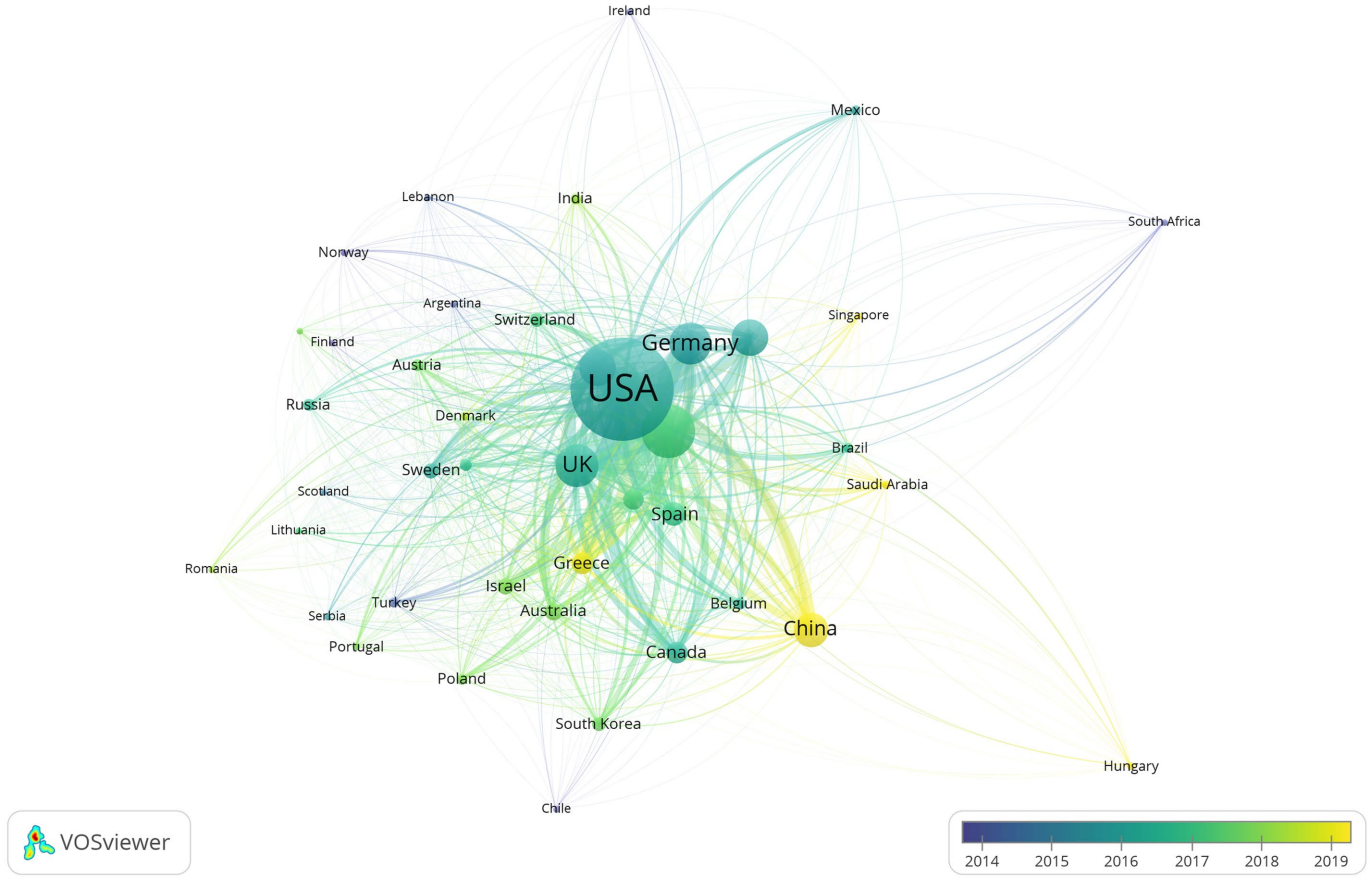
Network visualization of collaboration across countries chronologically in the field of AL amyloidosis.

The publication distribution of the top 20 countries/regions is shown in Figure 3. A total of 70 countries or regions have published articles in the AL amyloidosis field in the last 20 years. The United States (USA) has the highest number of publications (1,182 articles), followed by Italy with 395 articles, the United Kingdom (UK) with 278 articles, Germany with 266 articles, France with 215 articles, Japan with 214 articles, and China with 196 articles (Table 1). Among 70 countries, only 8 countries published more than 100 papers in the field. Additionally, the interconnection between countries is shown in Figure 4; Supplementary Figure S1. The size of the circle represents the number of articles published by the country. The shorter the distance between the two circles, the greater the cooperation between the two countries.

**Table 1:** Top 20 countries with a minimum of 5 published in the field of AL amyloidosis (61 countries found out of 98).

Country	Documents	Citations	Average citation	Total link strength
USA	1,182	46,900	39.68	34,766
Italy	395	21,718	54.98	21,431
UK	278	21,114	75.95	17,640
Germany	266	11,770	44.25	11,823
France	215	11,141	51.82	9,200
Japan	214	3,627	16.95	4,267
China	196	2,666	13.60	5,154
Spain	103	4,400	42.72	4,261
Greece	92	4,903	53.29	6,270
Canada	87	3,967	45.60	4,314
Netherlands	81	6,690	82.59	4,680
Australia	61	2,198	36.03	2,794
Israel	56	1,336	23.86	2,176
Sweden	50	3,002	60.04	1,525
South Korea	43	757	17.60	1,562
Switzerland	43	1,578	36.70	1,186
Austria	35	1,993	56.94	1,076
Belgium	32	1,654	51.69	1,619
Czech Republic	32	1,810	56.56	1,294
Russia	32	455	14.22	411

**Figure 5:**
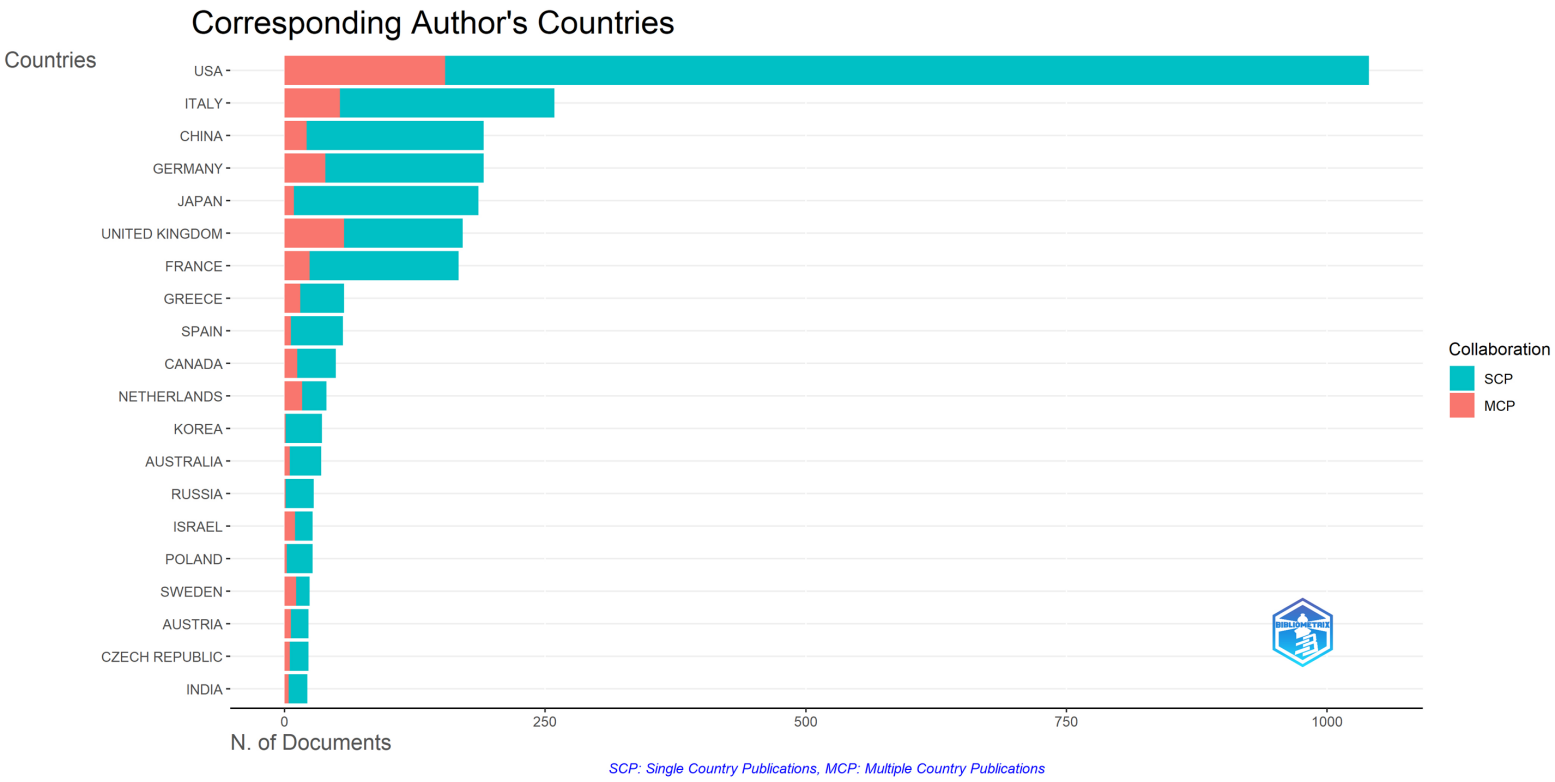
Inter and intra collaboration of various countries.

Moreover, the total link strength (TLS) can measure the collaboration across countries. The TLS analysis revealed that the USA had the highest collaborative research with a TLS of 34,766 for all 1,182 articles, Italian researchers ranked second with a TLS of 21,431, followed by the UK with a TLS of 17,640 (Table 1). Results also revealed that publications of the USA had the maximum number of citations (46,900 citations, with an average citation per document of 39.68), followed by Italy with 21,718 citations (average of 54.98), UK with 21,114 citations (average of 75.95) and Germany with 11,770 citations (average of 44.25). Figure 5 displays the intra and inter-collaboration of various countries to publish articles in the field of AL amyloid by analyzing single country publications (SCPs) and multiple country publications (MCPs). MCP represents collaboration among different countries, while SCP indicates the production of a single country. Countries were selected based on the corresponding author’s country. The USA and UK had the highest number of MCPs as compared to other countries, followed by Italy and Germany. The USA and Italy also had the first and 2nd highest number of SCPs, respectively (Figure 5).

### 3.3 Publication distribution of organization

**Figure 6:**
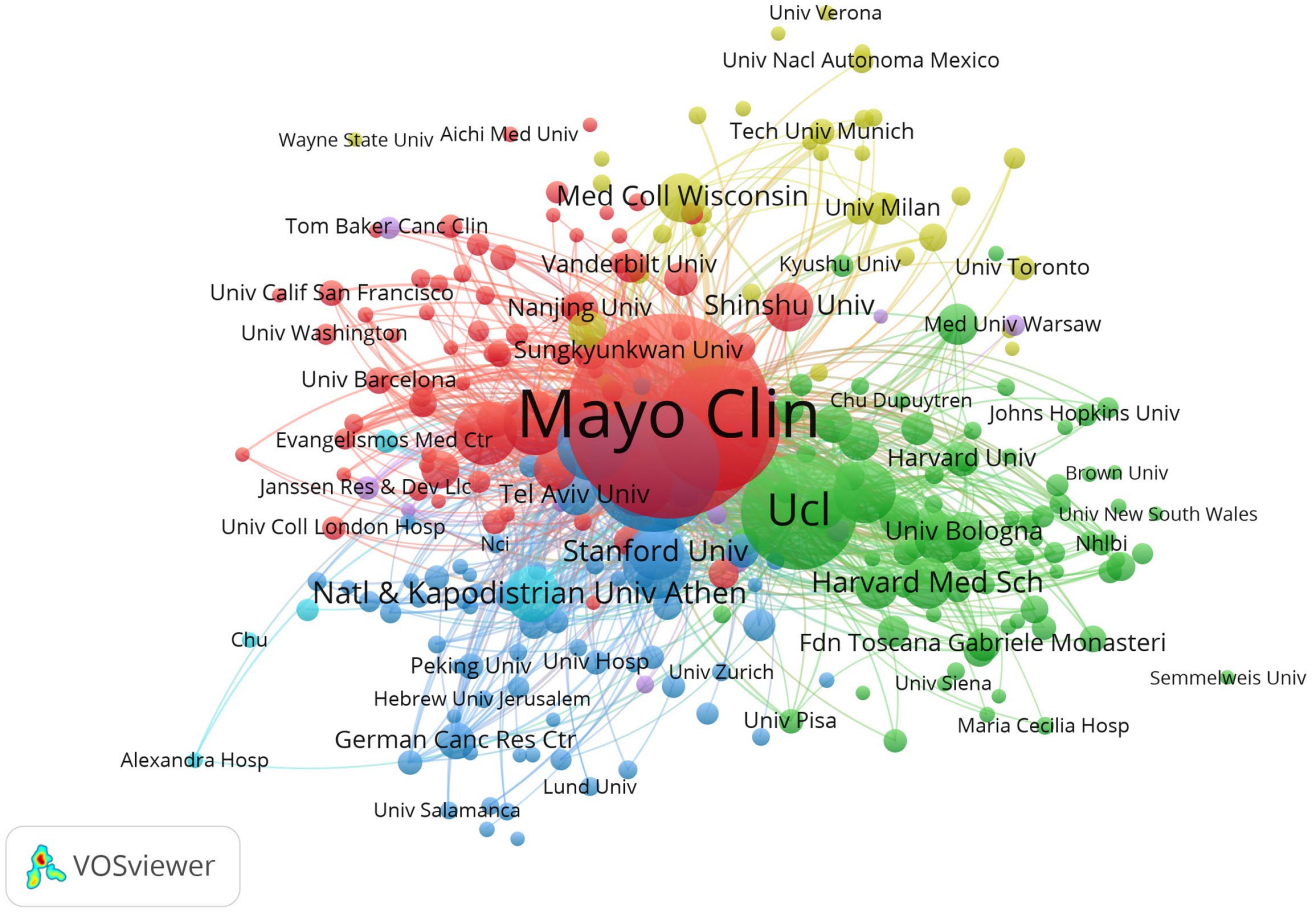
Organizations cooperation network map of relevant literature.

The organization with a minimum of 5 publications in the field of AL amyloidosis were analyzed using VOSviewer. A total of 2,896 institutions have participated in the studies of AL amyloidosis in the past 20 years, and 285 of them published more than 5 pieces of literature. The top five organizations with the highest number of publications were Mayo Clinic (394), University of Pavia (190), Boston University (175), University College London (153), and Fdn IRCCS Policlin San Matteo (69). The top 20 organizations with the highest number of published documents and citations are shown in Table 2, and the institute’s cooperation network map is displayed in Figure 6; Supplementary Figure S2.

**Table 2:** Top 20 institutes with a minimum of 5 published in the field of AL amyloidosis (285 countries found out of 2,896).

Organizaiton	Country	Documents	Citations	Average citation
Mayo Clinic	The United States	394	21,820	55.38
University of Pavia	Italy	190	12,540	66
Boston University	The United States	175	11,270	64.4
University College London	The United Kingdom	153	12,438	81.29
Fdn IRCCS Policlin San Matteo	Italy	69	3,542	51.33
Heidelberg University	Germany	63	3,729	59.19
Boston Medical Center	The United States	62	3,252	52.45
Memorial Sloan Kettering Cancer Center	The United States	57	3,553	62.33
Tufts Medical Center	The United States	56	3,264	58.29
Columbia University in the City of New York	The United States	55	4,536	82.47
National and Kapodistrian University of Athens	Greece	46	699	15.20
Stanford University	The United States	45	1,383	30.73
Harvard Medical School	The United States	40	1,017	25.43
Brigham and Women’s Hospital	The United States	38	3,965	104.34
Medical College of Wisconsin	The United States	37	762	20.59
Shinshu University	Japan	37	705	19.05
The University of Tennessee	The United States	32	1983	61.97
Cleveland Clinic	The United States	29	1,464	50.48
University of Athens	Greece	29	3,068	105.79
Kumamoto University	Japan	28	905	32.32

### 3.4 Most productive authors and co-author relationship

**Figure 7:**
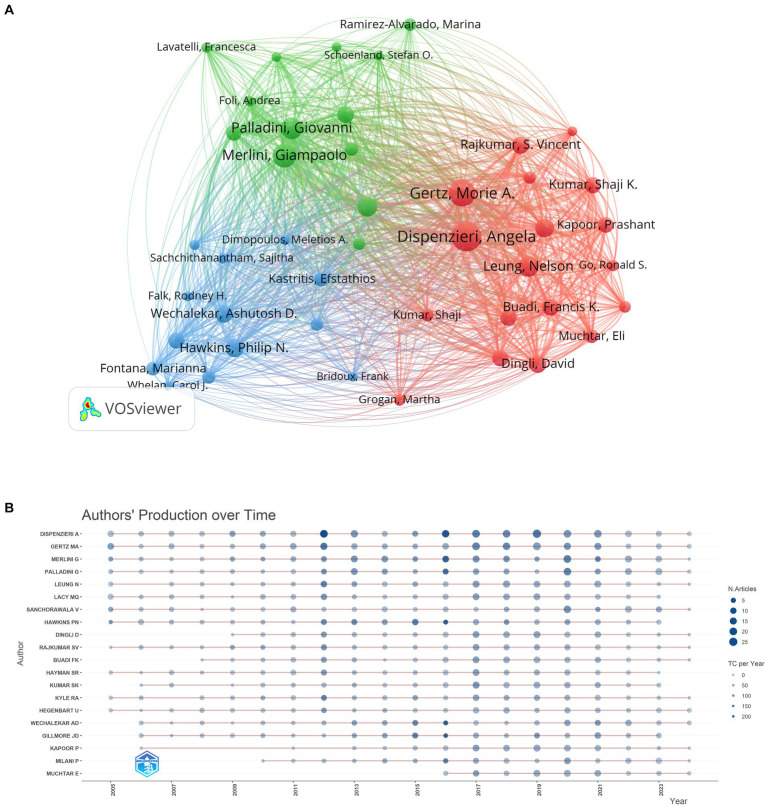
Author visual analysis. **(A)** Network visualization of collaborative research among authors. **(B)** Top 20 authors over different years.

Among 12,366 authors, 44 leading authors published more than 30 publications in the field of AL amyloidosis. The top 20 productive authors were shown in Supplementary Figure S3A, while the top 20 cited ones were displayed in Supplementary Figure S3B. According to the total number of publications, Dispenzieri A. (199), Gertz M. A. (165), and Merlini G. (135) were the top 3 most productive authors, respectively. In terms of the metrics of citations received, Dispenzieri A. (10,529), Merlini G. (9,080), and Hawkins P. N. (9,035) were the top 3 most productive authors, respectively (Table 3). *H*-index and *G*-index were used to characterize the scientific output of a researcher. The top 20 authors are all high impact with *H*-indexes over 30. Notably, Dispenzieri A. has the highest *H*-index of 65, and Merlini G. has the highest *G*-index of 117 (Supplementary Figures S3C,D). Analysis of the author’s cooperation network map is displayed in Figure 7A and Supplementary Figure S4. Moreover, the author’s production over time was analyzed to investigate the spatial and temporal progress of AL amyloidosis research. Figure 7B shows the contribution of the top 20 authors over different years. The size of the dots represents the number of articles, and the color of the dots (light to dark) represents the total citations (TC) per year.

**Table 3:** Leading authors in the field of AL amyloidosis.

Author name	Documents	Citations	Average citation	Total link strength
Dispenzieri A.	199	10,529	52.9095	17,441
Gertz M. A.	165	8,953	54.2606	18,772
Merlini G.	135	9,080	67.2593	17,576
Palladini G.	119	6,618	55.6134	16,487
Leung N.	110	5,484	49.8545	10,077
Sanchorawala V.	101	3,509	34.7426	7,874
Lacy M. Q.	91	4,325	47.5275	10,036
Hawkins P. N.	86	9,035	105.0581	8,178
Dingli D.	80	3,303	41.2875	8,828
Rajkumar S. V.	80	4,263	53.2875	8,271
Wechalekar A. D.	79	5,587	70.7215	7,670
Buadi F. K.	78	3,324	42.6154	9,027
Hegenbart U.	78	4,343	55.6795	8,024
Hayman S. R.	74	3,809	51.473	8,568
Gillmore J. D.	73	6,118	83.8082	5,512
Kumar S. K.	72	2,725	37.8472	8,078
Kyle R. A.	71	4,649	65.4789	7,365
Kapoor P.	69	1,874	27.1594	6,774
Milani P.	68	2,900	42.6471	9,228
Kastritis E.	59	2,831	47.9831	6,703

### 3.5 Primary academic journals

**Figure 8:**
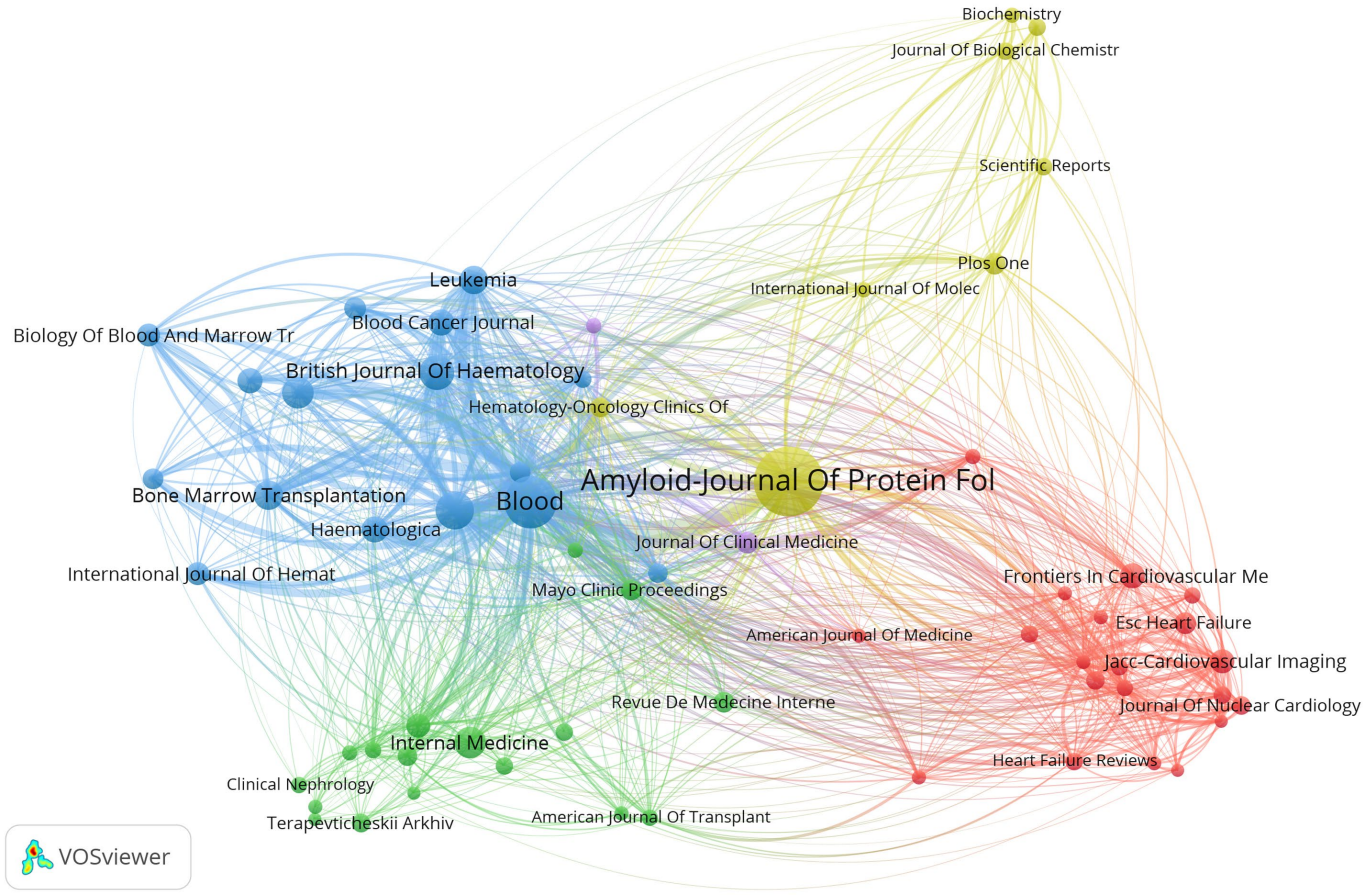
Primary journals visual analysis.

All the articles related to AL amyloidosis were published in 733 journals, among which 63 journals with a minimum number of 10 articles were selected for analysis (Figure 8; Supplementary Figure S5). Table 4 provides a list of the top 20 most productive journals that have published research articles in the field of AL amyloidosis. A total of 152 publications ranked the journal Amyloid-Journal of Protein Folding Disorders at the top position, followed by Blood (95), American Journal of Hematology (57), and British Journal of Haematology (48) (Supplementary Figure S6A). Among them, Blood (IF = 20.3) is the most cited journal with citations of 9,384. The journals’ production over time of top five most productive journals were shown in Supplementary Figure S6B.

**Table 4:** Top 20 journals with the highest number of publications.

Sources/Journals	Publisher	Documents	Citations	Average citation	Impact factor (2023)
Amyloid-Journal of Protein Folding Disorders	Taylor & Francis	152	2,567	16.89	5.5
Blood	American Society of Hematology	95	9,384	98.78	20.3
American Journal of Hematology	Wiley	57	2,673	46.89	12.8
British Journal of Haematology	Wiley	48	1,207	25.15	6.5
Clinical Lymphoma Myeloma & Leukemia	Elsevier	41	397	9.68	2.7
Internal Medicine	Japanese Society of Internal Medicine	40	251	6.28	1.2
Bone Marrow Transplantation	Springer	37	1,147	31	4.8
Leukemia	Springer	35	2,707	77.34	11.4
Blood Cancer Journal	Springer	31	721	23.26	12.8
Haematologica	Ferrata Storti Foundation	31	1,447	46.68	10.1
European Journal of Haematology	Wiley	29	366	12.62	3.1
Frontiers in Cardiovascular Medicine	Frontiers	28	116	4.14	3.6
JACC-Cardiovascular Imaging	Elsevier	27	2,630	97.41	14
Nephrology Dialysis Transplantation	Oxford University Press	26	673	25.88	6.1
Biology of Blood and Marrow Transplantation	Elsevier	25	405	16.20	4.3
International Journal of Hematology	Springer	24	152	6.33	2.1
ESC Heart Failure	Wiley	23	244	10.61	3.8
Journal of Clinical Medicine	MDPI	23	57	2.48	3.9
Leukemia & Lymphoma	Informa Healthcare	22	332	15.09	2.6
PLoS One	Public Library of Science	22	496	22.55	3.7

### 3.6 Documents co-citation and references burst analysis

**Figure 9:**
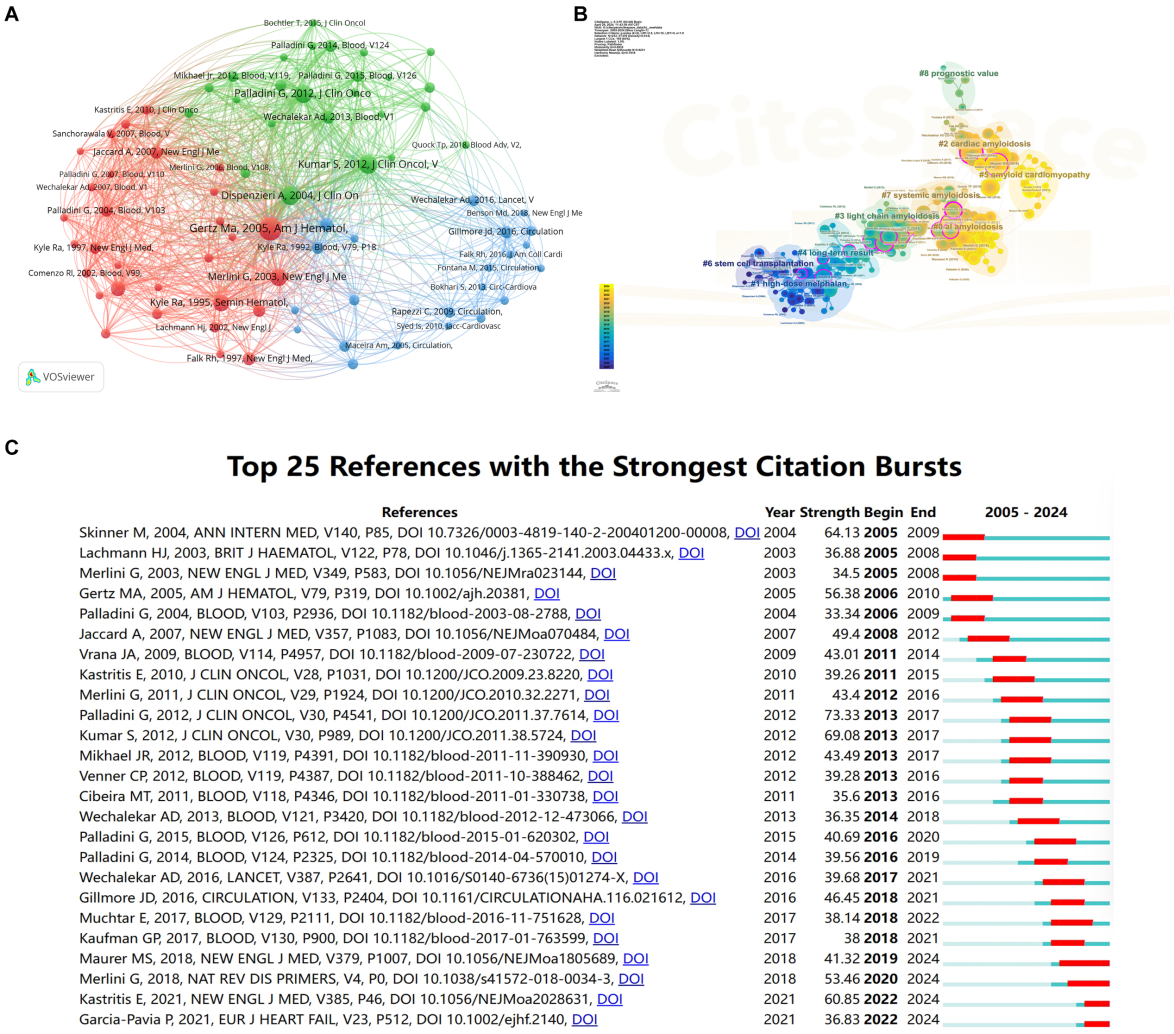
Visual analyses of AL amyloidosis documents and relevant cited references. **(A)** Analysis of documents citation. **(B)** Analysis of references clusters. **(C)** The top 25 references that had the most significant bursts of citations.

We selected articles with a minimum of 100 citations for analysis. Among the 39,672 documents, 77 articles meet the threshold (Figure 9A). The article Gertz (2005), American Journal of Hematology (13) has received the highest number of 695 citations. The 2nd and 3rd highest number of citations were received by papers authored by Kumar (2012), Journal of Clinical Oncology (14) and Dispenzieri (2004), Journal of Clinical Oncology (15) with 494 and 492 citations, respectively. Reference co-occurrence analysis generated a visual network map, which contains 233 nodes, 378 connected lines, and 9 main clusters (Figure 9B). The log-likelihood ratio (LLR) was used to characterize the nature of a cluster by CiteSpace, and then noun phrases were extracted from the keywords in the cluster. Log-likelihood ratio (LLR) clustered the cited literature into 9 groups, with the first three clusters’ labels named (#0) AL amyloidosis, (#1) high-dose melphalan, and (#2) cardiac amyloidosis, respectively. The weighted mean silhouette was 0.9231, which indicates the structure of the clustering has good stability and credibility. The modularity *Q* is 0.6932, which indicates that the clustering of the network is reasonable. The top 25 references with the strongest citation bursts are displayed in Figure 9C, which shows the first citation bursts occurred in 2005 (16). The blue bars represent the timeline, and the red bars represent the start and end years of the burst duration. An article entitled “New Criteria for Response to Treatment in Immunoglobulin Light Chain Amyloidosis Based on Free Light Chain Measurement and Cardiac Biomarkers: Impact on Survival Outcomes” authored by Palladini G. and colleagues published in Journal of Clinical Oncology (Q1, IF: 45.3) in 2012 (17) was the strongest citation burst with a strength value of 73.33 during a period of 4 years until 2017.

### 3.7 Analysis of burst words, keyword clusters and co-occurrence network

**Figure 10:**
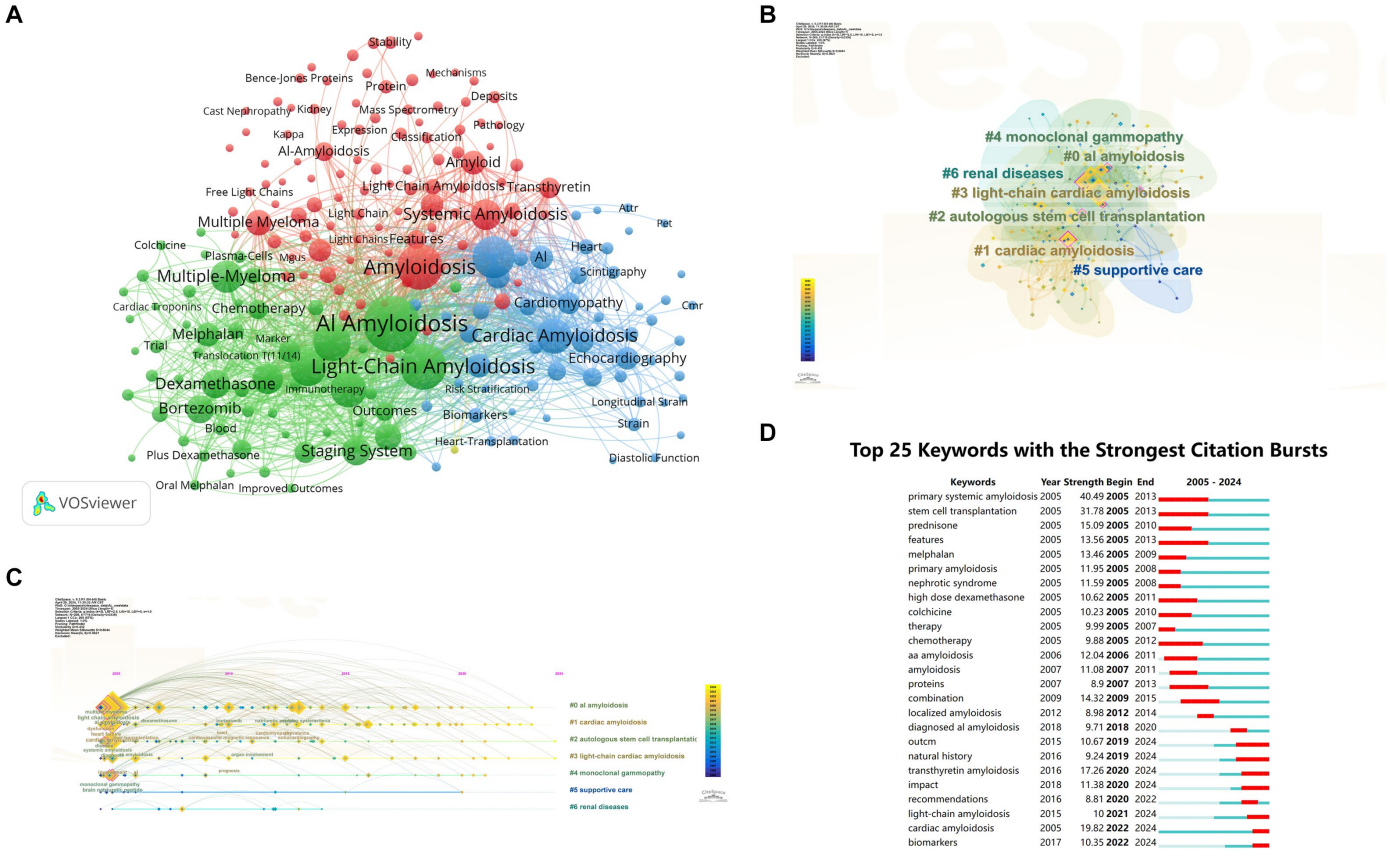
Analyses of keywords. **(A)** Visual analysis of keywords co-occurrence. **(B)** Cluster view of co-citation of keywords. **(C)** A timeline view of the co-citation of keywords. **(D)** The 25 most cited keywords.

A total of 6,147 keywords were obtained, while keywords with a minimum of 20 occurrences were taken into consideration. Keywords co-occurrence analysis revealed a total of 216 keywords had a minimum of 20 occurrences. Among them, the term “AL amyloidosis” had the highest frequency with 1,048 occurrences, followed by “Light-Chain Amyloidosis” with 715 occurrences, and “Amyloidosis” with 701 occurrences (Figure 10A; Supplementary Figure S7). The keyword clustering analysis was conducted in CiteSpace. Results showed the hotspots in the field of AL amyloidosis. As shown in Figure 10B, keywords were grouped into seven clusters, (#0) AL amyloidosis, (#1) cardiac amyloidosis, (#2) autologous stem cell transplantation, (#3) light-chain cardiac amyloidosis, (#4) monoclonal gammopathy, (#5) supportive care, and (#6) renal diseases, respectively. The timeline of keywords further elucidated the temporal characteristics and evolution trend of the core terms (Figure 10C). Additionally, the top 25 keywords with the strongest citation bursts based on citation burst values were identified, including primary systemic amyloidosis, stem cell transplantation, prednisone, features, melphalan, primary amyloidosis, nephrotic syndrome, high dose dexamethasone, colchicine, therapy, chemotherapy, aa amyloidosis, proteins, combination, localized amyloidosis, diagnosed AL amyloidosis, outcome, natural history, transthyretin amyloidosis, impact, recommendations, light-chain amyloidosis, cardiac amyloidosis and biomarkers (Figure 10D).

### 3.8 Three-field plot analysis representing keywords-sources-countries

**Figure 11:**
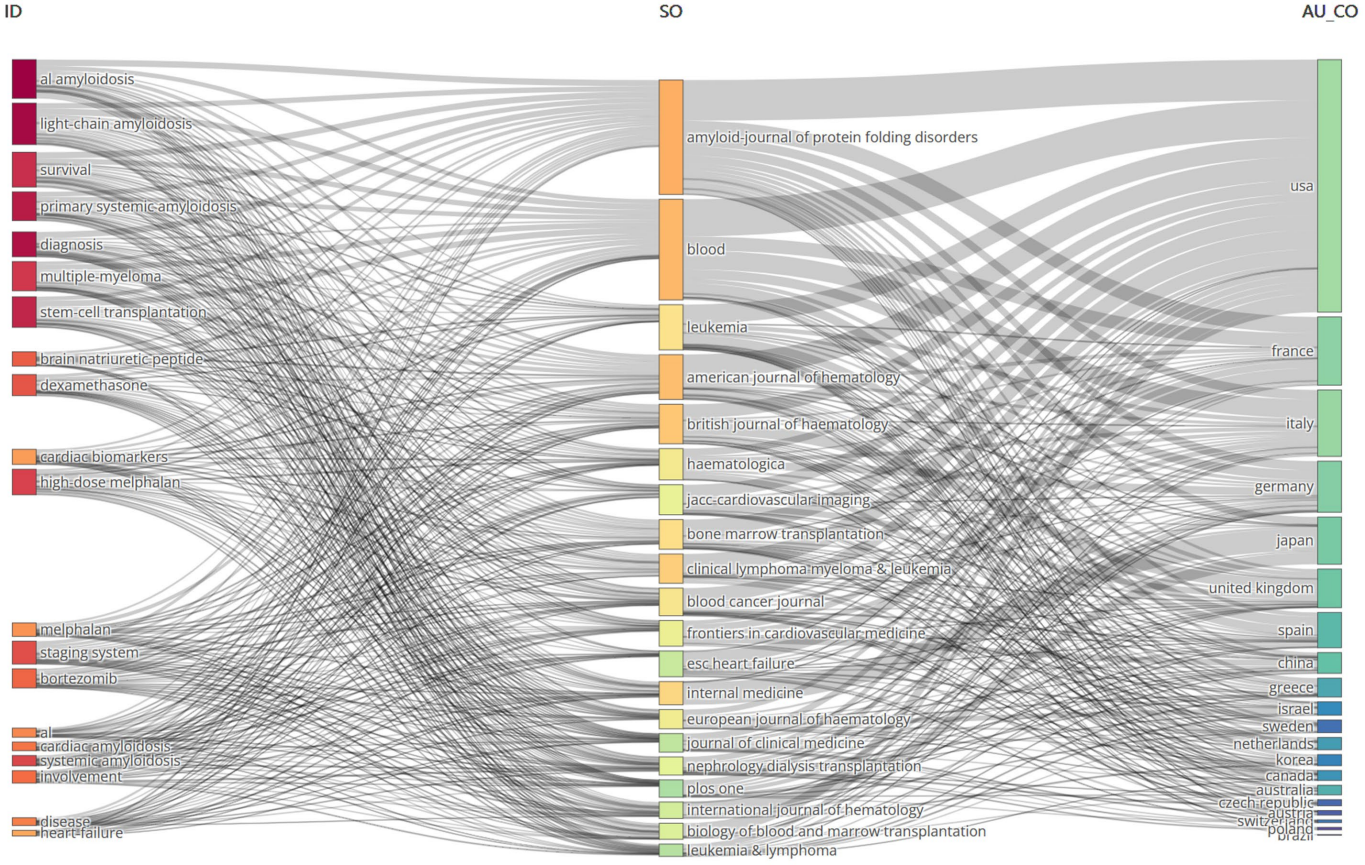
The three-field plot analysis representing keywords (left), sources (middle), and countries (right).

The three-field plot displayed the relationship between keywords, sources and countries (Figure 11). The lines and boxes demonstrate the association between the three variables. The height of the boxes and the thickness of the connecting lines show the strength of the association between variables (18). The result highlights “AL amyloidosis” as the most prevalent keyword connected strongly with the source Amyloid-journal of protein folding disorders. Moreover, the source Amyloid-journal of protein folding disorders had highly strength of association with the keywords “AL amyloid,” “light-chain amyloidosis,” “survival,” and “primary systemic amyloidosis” and countries like the USA, France and Italy.

## 4 Discussion

### 4.1 Basic information

This bibliometric study was conducted to identify the status of global scientific output, the hotpots, frontiers, and future trends of AL amyloidosis between January 1, 2005 and April 28, 2024. Software including CiteSpace, VOSviewer, and R were applied to analyze and visualize the countries/regions, authors, organizations, and sources of documents published over the past 20 years. A total number of 4,980 publications was retrieved from the WOSCC database. According to the screening criteria, 2,864 papers (including 2,277 articles and 587 reviews) met the threshold, which were written by 12,366 authors from 2,896 institutions in 70 countries/regions, published in 733 journals with 39,672 co-cited references.

A steady increasing trend in the number of international publications indicates that this field is a promising field, which continues to attract the attention of international researchers. Overall, the United States and some developed countries dominate the international research in the AL amyloidosis field. The United States had the highest number of documents, citations and total link strength, followed by Italy, the United Kingdom, Germany, and France, which implies that The United States dominates the core of the field and has formed academic cooperation networks with many European countries. There are 12 American organizations among the top 20. It is necessary for the above-mentioned countries to take an active lead in establishing worldwide academic cooperation networks. Some Asian countries like Japan, China, South Korea, Saudi Arabia and Singapore, as shown in Table 1 and Figure 4, have started paying attention to this field in recent years as well. From 2015 to 2023, the number of publications in this field increased significantly, and to date, 48 papers have been published in 2024, indicating that AL amyloidosis has been attracted increasingly attention in recent few years and is undergoing a rapid developmental stage.

### 4.2 Hotspots and frontiers

Keyword frequency analysis, co-occurrence network mapping, and cluster analysis serve as valuable tools for identifying the research themes and hotspots in AL amyloidosis over the past two decades, providing crucial guidance for researchers. In Figure 10A, keywords are grouped into 3 clusters, delineating the pathological mechanisms of light chain amyloid protein formation (red), clinical diagnosis and treatment strategies for AL amyloidosis (green), and cardiac-related amyloidosis (blue). Burst keywords and references highlight prominent research directions. Among the top 25 burst keywords and references in CiteSpace software, it reflects that the researchers focus on the clinical efficacy of treatment on AL amyloidosis and the diagnosis in cardiac amyloidosis. We will further elaborate on the following 3 research directions.

#### 4.2.1 Biomarkers

Appropriate biomarkers play a crucial role in the early diagnosis and prognostic assessment of AL amyloidosis due to its non-specific presentation, which leads to delayed diagnosis, and increased mortality rates (19). However, a significant challenge persists in identifying a sensitive and specific biomarker that encompasses all aspects of disease, including cardiac damage, renal failure, monoclonal plasma cell burden, and toxic free immunoglobulin light chains circulation (19).

Given the significant prognostic implications of cardiac involvement and its association with early mortality, biomarkers indicative of cardiac injury and dysfunction have emerged as powerful prognostic indicators over time. The Mayo 2004 staging system, an early benchmark, incorporates three biomarkers: N-terminal pro-brain natriuretic peptide (NT-proBNP), brain natriuretic peptide (BNP), and cardiac troponin T (cTnT) (15). Subsequent refinements, such as the Mayo 2012 system, introduced additional parameters like the difference between involved and uninvolved free light chain concentration (dFLC), recognizing the significance of FLC levels in reflecting the underlying clonal disease burden (14). Although the combination of cTnT and NT-proBNP offers an objective and reproducible risk assessment tool, their reliability may be compromised in the presence of renal dysfunction and other confounding factors, such as fluid overload and atrial arrythmias (20). Hence, their reliability in such contexts is therefore questioned. The current staging system incorporates estimated glomerular filtration rate (eGFR) and proteinuria for prognostic evaluation of renal involvement, serving as endpoints in clinical trials (21). However, neither marker is sufficiently specific nor sensitive, nor do they directly reflect renal cell injury. Even though creatinine, eGFR, and proteinuria are established biomarkers, they are subject to influences such as hydration status, diuretic use, fluctuations in weight, and comorbidities, necessitating ongoing efforts to identify more sensitive biomarkers (19).

In addition to the abovementioned biomarkers, other parameters including cardiac troponin I (cTnI) (22), high-sensitivity cardiac troponin(hs-cTnT) (23), ejection fraction (EF) (24), left ventricle longitudinal function (25), left ventricle septum thickness (15), systolic blood pressure (26), uric acid (27), albumin-to creatinine ratio (28), albumin (21), and proteinuria (21) have also been explored as indicators in patients with AL amyloidosis in recent years.

Tumor-related biomarkers were screened based on the characteristics of the underlying clonal disorder, the burden and qualities of the clonal plasma or other B-cells, and the secreted amyloidogenic FLCs. They have emerged as indicators predominantly associated with long-term prognostic outcomes, in contrast to organ-function-related markers which tend to predict short-term outcomes. Furthermore, they hold significant value in various clinical aspects including response assessment, patient surveillance, and tailoring of treatment. In this respect, quantity of serum FLC (29), bone marrow plasma cell burden (30), abnormality in cytogenetics of the plasma cell clone (31) and immunoparesis (32) serves as adverse factors in AL amyloidosis prognosis.

Novel biomarkers have emerged as potential tools for refining risk stratification and predicting outcomes in the AL amyloidosis population. These include D-dimer (33), von Willebrand factor (vWF) (34), red cell distribution width (RDW) (35), soluble suppression of tumorigenicity 2 (sST2) (36), osteopontin (37), flow-mediated dilatation (FMD) (38), myocardial contraction fraction (39), left ventricle longitudinal axis strain (39), extracellular volume (ECV) (40), growth differentiation factor-15 (GDF-15) (41), soluble urokinase-type plasminogen receptor (suPAR) (42), and galectin-3 (Gal-3) (43). However, their clinical utility remains to be verified. Given the complexity and heterogeneity of the disease, it is unlikely that a single biomarker will adequately reflect prognostic value such as survival, risk of dialysis, and treatment response. With the advent of new diagnostics and therapeutics, there is potential for the discovery of additional biomarkers. On one hand, there is a need to conduct largescale epidemiological surveys and multicenter clinical trials to obtain detailed and complete patient information. On the other hand, with the era of big data, artificial intelligence should be used to in biomarker discovery.

#### 4.2.2 Cardiac amyloidosis

Cardiac amyloidosis is characterized by the extracellular deposition of misfolded proteins known as amyloid fibrils within the myocardium, typically exhibiting Congo red positive staining and green birefringence when viewed under cross polarized light microscopy. Among the 9 amyloidogenic proteins accumulating in the myocardium to cause significant cardiac disease, the most prevalent are monoclonal immunoglobulin light chain (AL amyloidosis) and transthyretin (ATTR) (44). AL cardiac amyloidosis often eludes timely diagnosis or is misdiagnosed due to its non-specific clinical manifestations and relative rarity (45). Differential diagnosis challenges may arise, such as distinguishing between cardiac amyloidosis, hypertrophic cardiomyopathy, or heart failure. The identification of individuals with pre-symptomatic AL amyloidosis prior to the onset of significant organ involvement may allow for facilitating more efficacious therapeutic interventions, resulting in improved response rates and survival.

The diagnosis of cardiac amyloidosis involves two distinct phases: (i) the suspicious phase, and (ii) the definitive diagnosis phase. During the suspicious phase, extracardiac signs and symptoms like polyneuropathy, dysautonomia, skin bruising, macroglossia, renal insufficiency and proteinuria, in conjunction with cardiac indications such as hypotension or normotensive (in previously hypertensive patients), pseudoinfarct pattern, low or decreased QRS voltage to degree of left ventricular thickness, atrio-ventricular conduction disease, disproportionally elevated NT-proBNP to degree of heart failure, persistent elevation in troponin levels, granular sparkling of the myocardium, increased right ventricular wall thickness, increased valve thickness, pericardial effusion, and reduced longitudinal strain with apical sparing pattern, should raise suspicion for cardiac amyloidosis. Both invasive and non-invasive diagnostic modalities are available for cardiac amyloidosis, whereas non-invasive criteria are primarily applicable to transthyretin amyloidosis (ATTR), such as Grade 2 or 3 cardiac uptake at diphosphonate Scintigraphy combining with typical echocardiographic/cardiac magnetic resonance (CMR) findings and negative serum free light chains together with negative serum and urine immunofixation (46, 47). The presence of Congo red-positive deposits on biopsy remains the gold standard for the diagnosis of amyloidosis. Subsequently, accurate typing of the amyloid is critical to guide specific treatment strategies. Mass spectrometry stands as the gold standard for determining the amyloid type (48).

While several staging systems in cardiac amyloidosis have been put forward to facilitate prognosis, there are limited data on how to assess progression (47). This field requires urgent efforts from clinician and researchers. Treatment of cardiac amyloidosis involves supportive care, and specific treatment, such as targeting the production of amyloid precursor protein or the aggregation of amyloid fibrils. Tafamidis has shown efficacy in a randomized trial in patients with wild-type transthyretin and variant transthyretin amyloidosis with cardiomyopathy (49).

#### 4.2.3 Treatment

In the mid-1990s, high-dose melphalan and autologous stem cell transplantation (HDM/SCT) demonstrated positive survival outcomes, and make it a crucial standard of care for early-diagnosed patients (50). Subsequently, in the mid-2000s, oral melphalan with dexamethasone became the standard of care for non-transplant-eligible patients (51). Bortezomib came into view as a third-generation treatment option. The marked excess of misfolded toxic light chains in AL renders plasma cells more sensitive to proteasome inhibition in AL compared to multiple myeloma (52). The combination of bortezomib-dexamethasone with cyclophosphamide (VCD) has shown very good partial responses in more than half of all patients, and complete responses in a quarter of patients (53). However, the recognized cardiac toxicity of proteasome inhibitors raises concerns about their contribution to early deaths in AL. Daratumumab, a high-affinity human IgGκ1 monoclonal antibody targeting CD38, is an antigen ubiquitously expressed all plasma cells, causing cell death by multiple pathways (6). Thus, it reduces the concentration of monoclonal immunoglobulin light chain quickly. Daratumumab has shown efficacy in relapsed AL amyloidosis and is being investigated for in front-line treatment. Clinical trials combining daratumumab with ixazomib, pomalidomide, bortezomib, and dexamethasone are currently underway. However, the limitation of daratumumab lies in its increased risk of infections (54), necessitating large-scale randomized experiments to validate its impact. Despite advancements in therapeutic method leading to gradual improvements in survival rates, little progress has been made in treating advanced patients, and addressing the actual deposits remains challenging (54). New therapies, such as anti-CD38 antibody isatuximab, chimeric antigen receptor T cells, bispecific T-cell engager antibodies, and antibody-drug conjugates, are emerging. Future researches should focus on the drug efficacy through international collaborative clinical trials. Combination anti-plasm cell therapy with above-mentioned new therapies will offer a more complete approach to cure this disease, however, the most reasonable joint scheme, the optimal administration sequence and the safety are directions that need to be explored (55). Additionally, while keywords such as “aggregation,” “fibril formation” and “protein aggregation” experienced a burst in interest 5 years ago, continued attention to basic and translational research on the aggregation and deposition mechanisms of immunoglobulin light chain is warranted, as the underlying mechanisms can provide a theoretical basis for clinical application and new drug development. In a word, the treatment of AL amyloidosis requires comprehensive concept in the future, which still calls for continuous basic and translational research.

## 5 Advantages and limitations

The current bibliometric investigation offers comprehensive analyses of scholarly outputs in the realm of AL amyloidosis spanning the period from 2005 to 2024, including publication volumes and growth patterns, primary sources, leading authors, collaborative networks, as well as bursts of keywords and references. We simultaneously employed three bibliometric tools, namely CiteSpace, VOSviewer and Biblioshiny package, improving the credibility and robustness of our findings. It is inevitable to acknowledge a limitation of our study, namely, the restriction of our literature research to the WOSCC database. Consequently, despite this database is widely recognized for its comprehensiveness and reliability, its coverage may not encompass all relevant publications on AL amyloidosis. There exists the possibility of overlooking valuable insights and perspectives offered by studies not included in our analysis.

## 6 Conclusion

This bibliometric analysis examined the research developments in AL amyloidosis over the past two decades using bibliometric software. A total of 2,864 relevant documents were retrieved from the Web of Science Core Collection. Research activity in the field of AL amyloidosis has exhibited a steady increase, with countries such as the United States and various European nations leading in achievements, consistent with the organizations and authors in the field. Notably, publications are predominantly found in journals specializing in amyloid research and hematology. The journal with the highest publication volume is Amyloid-Journal of Protein Folding Disorders. Recent research in this field primarily focuses on two main areas: clinical diagnosis and treatment of AL amyloidosis, as well as cardiac amyloidosis. Emphasis is placed on understanding the mechanisms underlying immunoglobulin light chain aggregation and deposition to mitigate organ involvement.

## Data availability statement

The original contributions presented in the study are included in the article/Supplementary material, further inquiries can be directed to the corresponding authors.

## Author contributions

XL: Data curation, Methodology, Software, Visualization, Writing – original draft. JW: Data curation, Methodology, Software, Visualization, Writing – review & editing. YL: Writing – review & editing. WS: Writing – review & editing. XZ: Writing – review & editing. SL: Conceptualization, Funding acquisition, Project administration, Supervision, Writing – review & editing. BC: Conceptualization, Funding acquisition, Project administration, Supervision, Writing – review & editing.

## Footnotes

^1^ https://www.bibliometrix.org
