# Supplementary material for: A bibliometric analysis of light chain amyloidosis from 2005 to 2024: research trends and hot spots
Source: Front Med (Lausanne). 2024 Jul 30;11:1441032. doi: 10.3389/fmed.2024.1441032 (PMC11320149; doi:10.3389/fmed.2024.1441032)
Supplement: Supplementary file 4 [file Data_Sheet_4.docx]

Supplementary Material
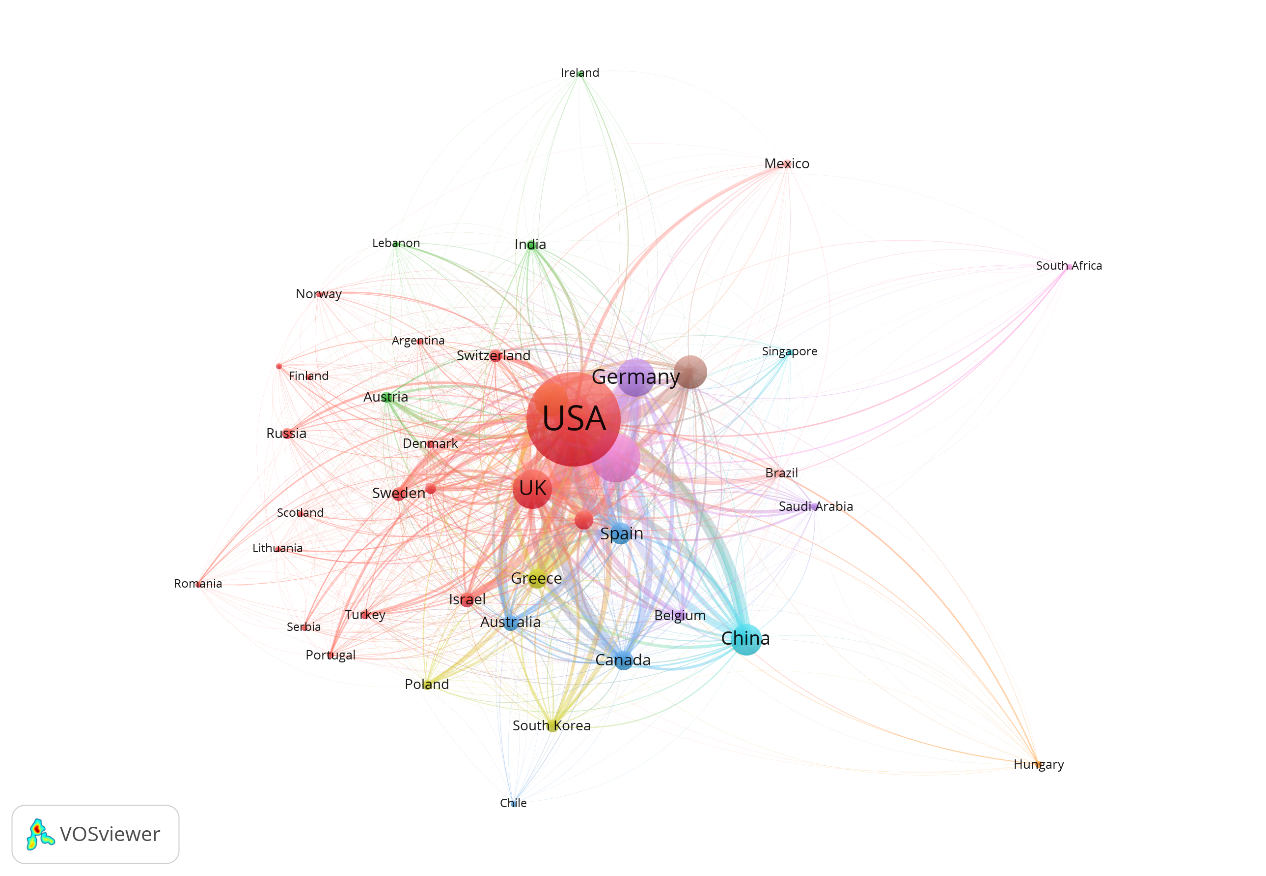


**Supplementary Figure 1.** Network visualization of collaboration across countries in the field of AL amyloidosis.

**
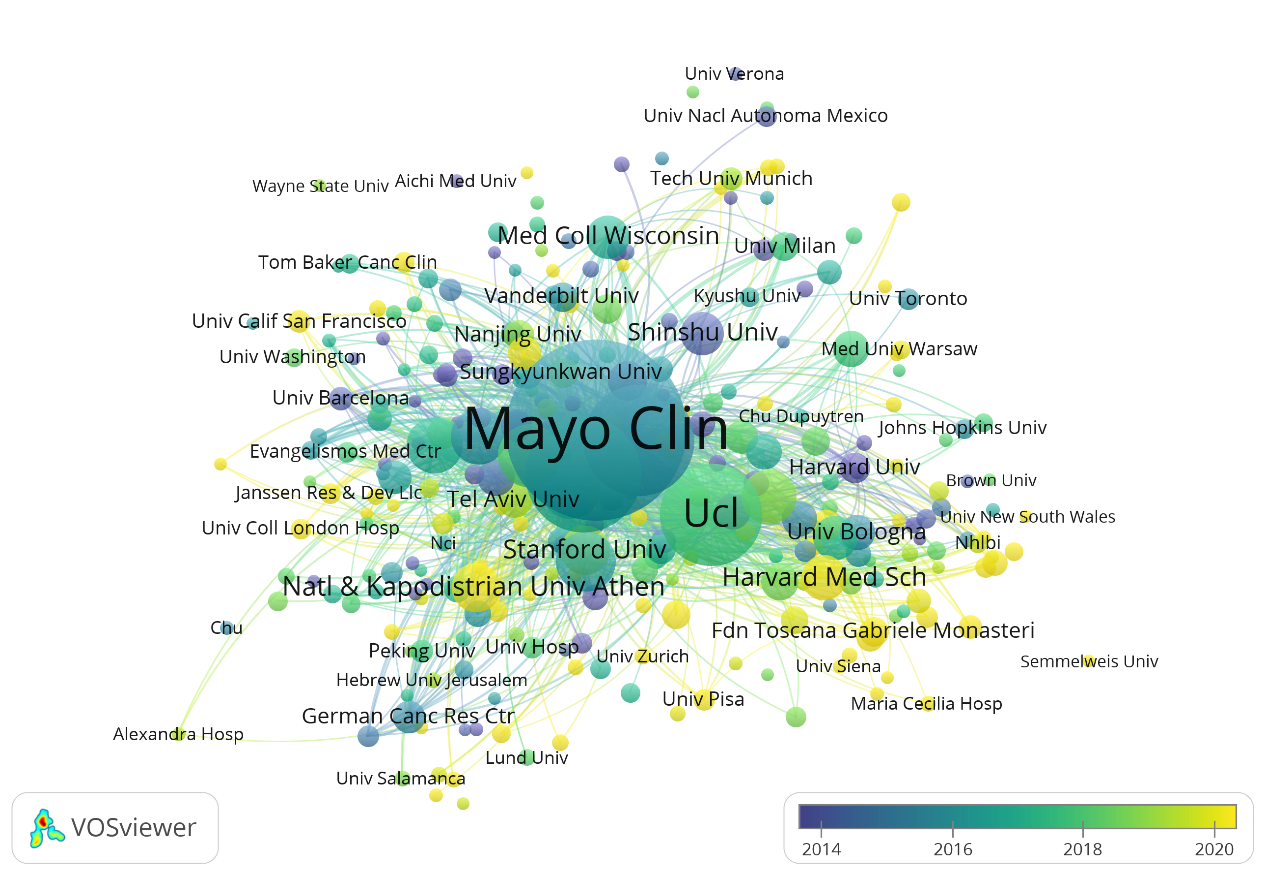
**

**Supplementary Figure 2.** Organizations cooperation network map of relevant literature chronologically in the field of AL amyloidosis.

**
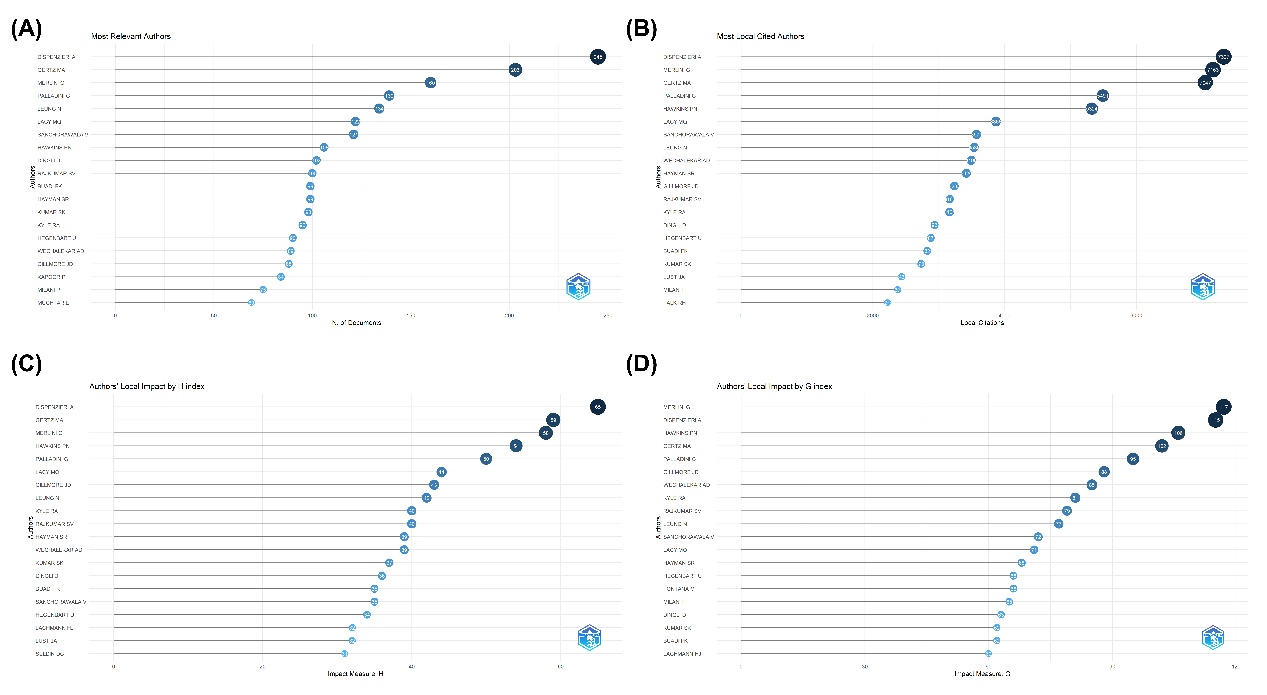
**

**Supplementary Figure 3.** The bibliometric analysis of the authors. (A) Top 20 most productive relevant authors in the field of AL amyloidosis; (B) Top 20 most local cited authors in the field of AL amyloidosis; (C) Top 20 authors’ local impact measured by H-index value; (D) Top 20 authors’ local impact measured by G-index value.

**
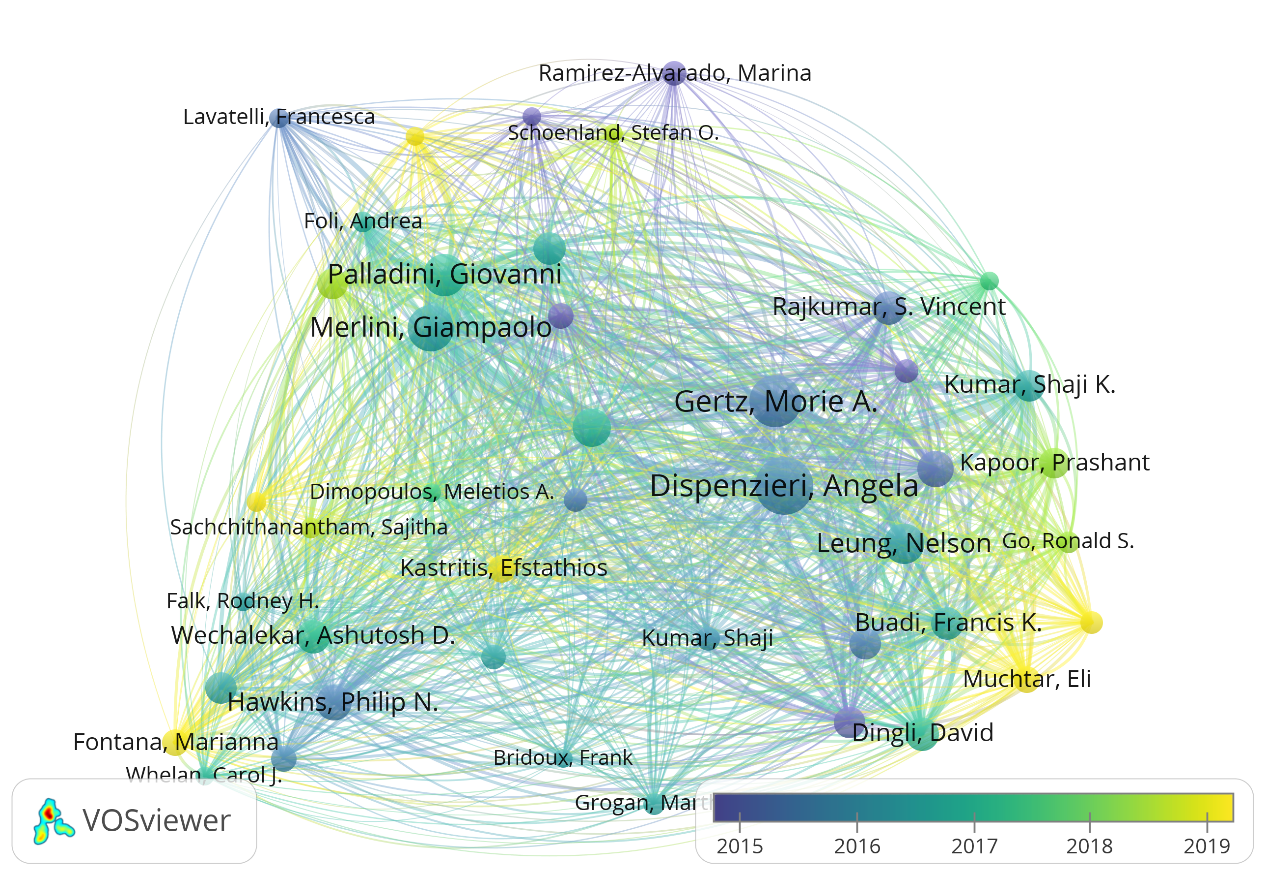
**

**Supplementary Figure 4.** Network visualization of collaborative research among authors chronologically in the field of AL amyloidosis.

**
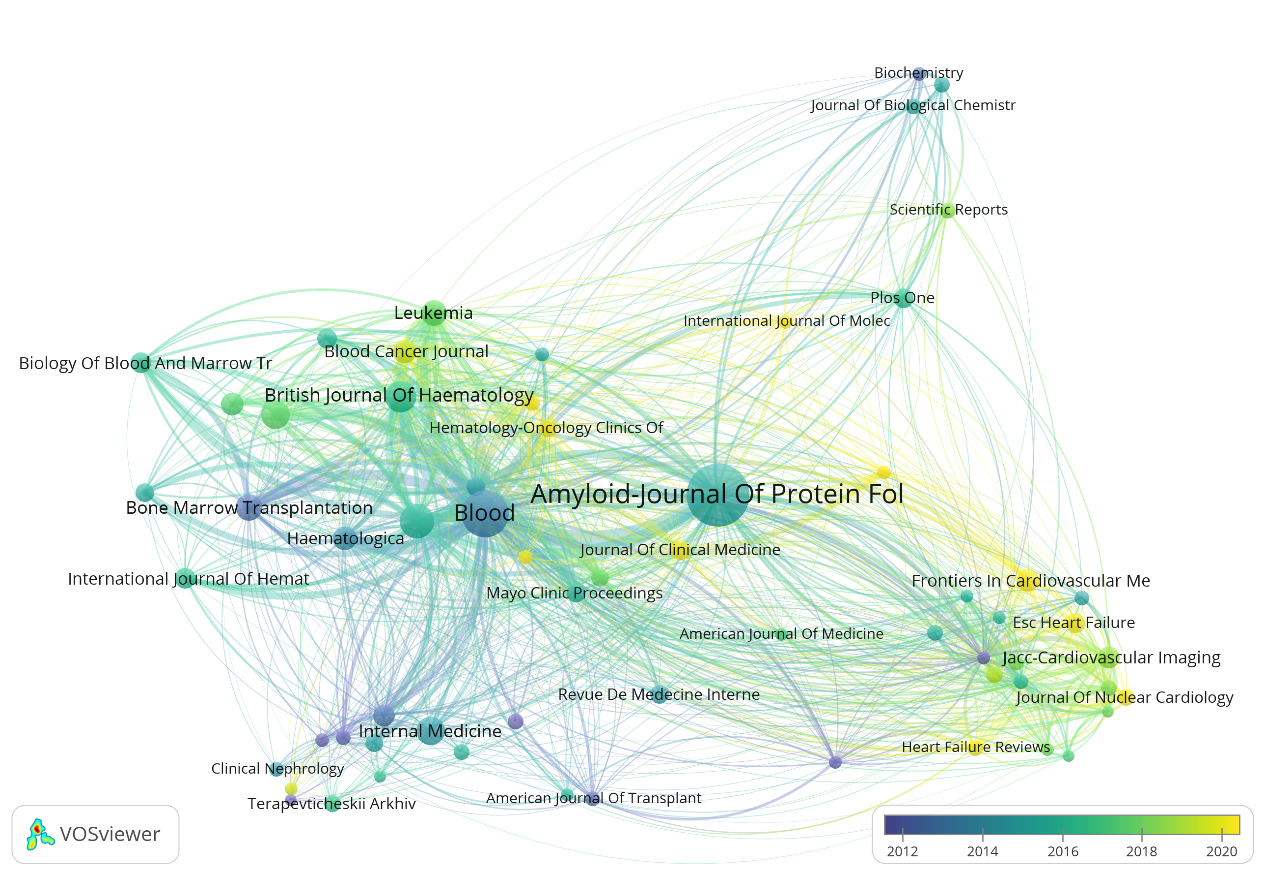
**

**Supplementary Figure 5.** Primary journals visual analysis chronologically in the field of AL amyloidosis.

**
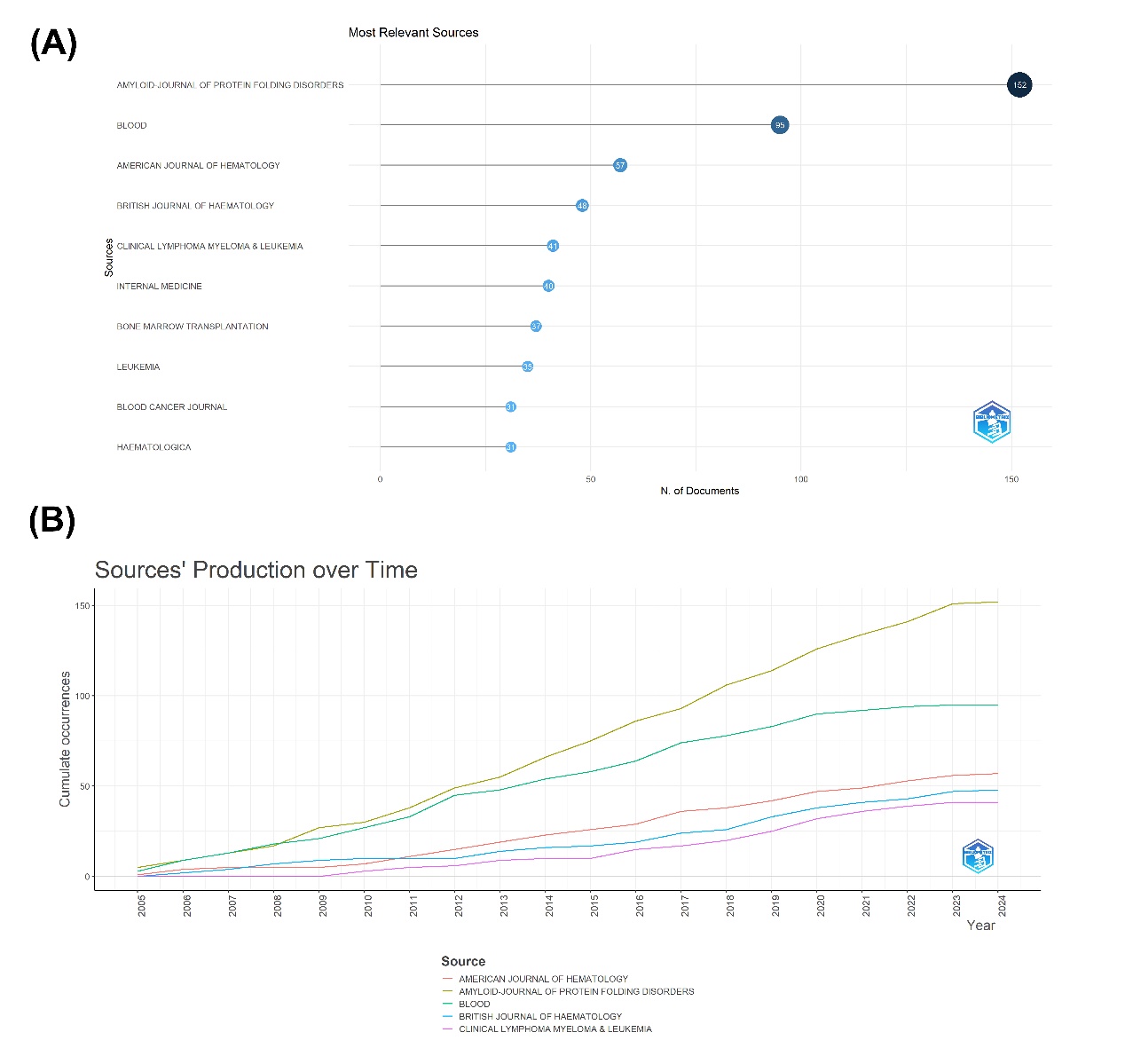
**

**Supplementary Figure 6.** Primary journals visual analysis. (A) Top 10 most relevant sources; (B) Top 5 most relevant sources’ production over time.

**
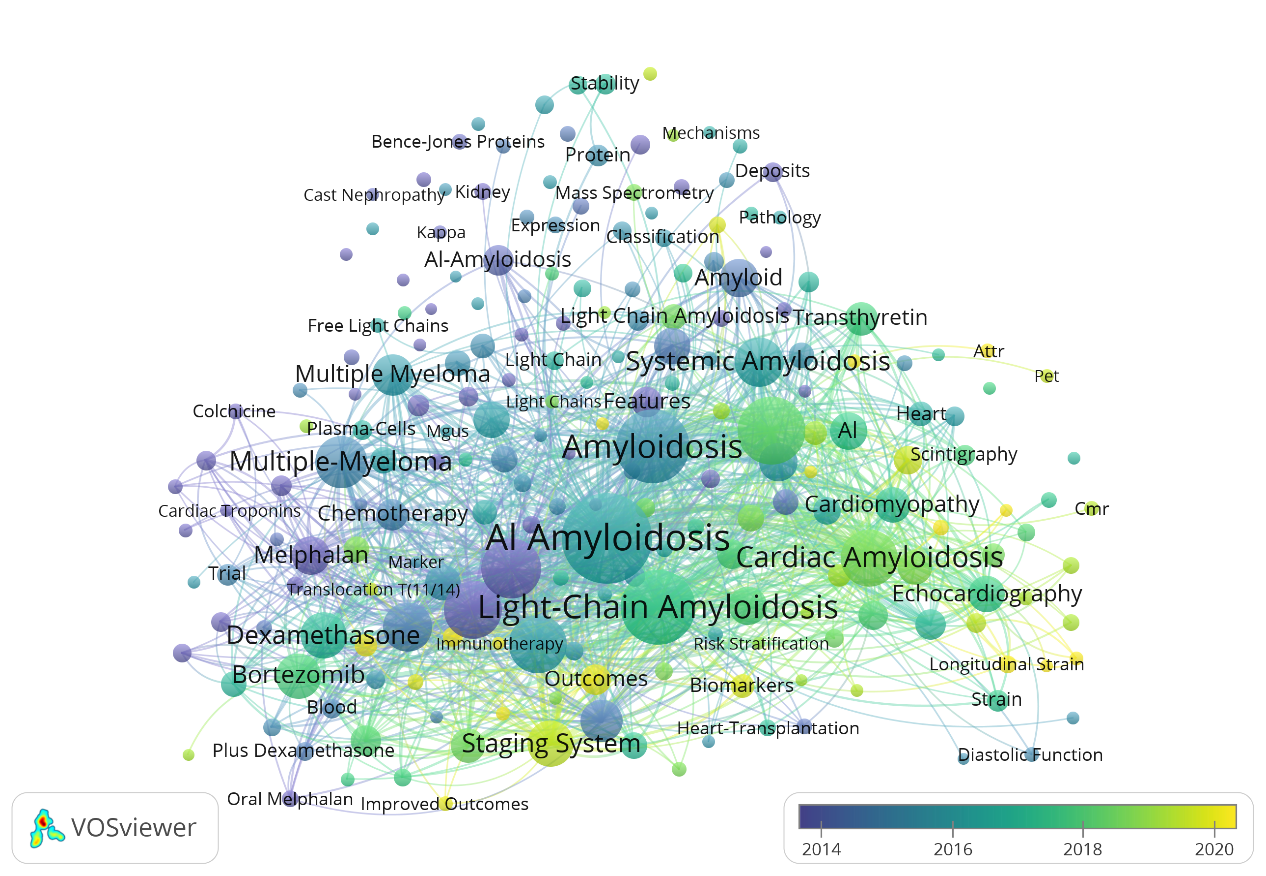
**

**Supplementary Figure 7.** Visual analysis of keywords co-occurrence chronologically in the field of AL amyloidosis.
